# Study of the magnitude of diabetes and its associated risk factors among the tuberculosis patients of Morang, Eastern Nepal

**DOI:** 10.1186/s12889-019-7891-x

**Published:** 2019-11-21

**Authors:** Babita Sharma, Vijay Kumar Khanal, Nilambar Jha, Prajjwal Pyakurel, Gyanu Nepal Gurung

**Affiliations:** School of Public Health and Community Medicine (SPHCM), B. P. Koirala Institute of Health Sciences (BPKIHS), Dharan, Nepal

**Keywords:** Diabetes, Tuberculosis, Co-morbidity, Prevalence, Eastern Nepal

## Abstract

**Background:**

WHO addresses the infectious disease like Tuberculosis, and non- communicable disease like Diabetes among the top 10 causes of death worldwide, which collectively leads to increasing mortality and premature death especially in developing countries. Hence, the present study aims to assess the prevalence of diabetes and its associated risk factors among the tuberculosis patient of Morang, Eastern Nepal.

**Methods:**

A cross-sectional study was carried out among the 320 respondents undergoing tuberculosis treatment of Morang district. Respondents from eight randomly selected DOTS centers were selected purposively. The Fasting Blood Sugar and 2-h Post-Prandial Blood Sugar were assessed in the laboratory of respective DOTS center by the glucose oxidase method. An interview for socio-demographic and other variables was conducted using a pretested semi-structured questionnaire based on WHO-STEP Instrument for chronic disease and excerpt from DASS-21 was used for the variable stress.

**Results:**

The prevalence of diabetes, pre-diabetic and glucose intolerance among tuberculosis patient was 11.9, 17.2, and 17.8% respectively. Additionally, the univariate analysis reported, user of tobacco products, current alcohol consumers, family history of diabetes and stress level, to have positive association with diabetes, while the multivariate analysis reported, the current alcohol consumer as the significant predictor of diabetes among the tuberculosis patient.

**Conclusion:**

A significant portion of the respondents were diabetic, impaired glucose tolerance and pre-diabetic, which supports the fact of diabetes being comorbid with tuberculosis. Hence, it shifts the focus on the bidirectional screening of tuberculosis and diabetes.

## Background

Diabetes mellitus (DM) is a serious lifelong condition, which occurs when the pancreas does not produce enough insulin, or when the body cannot effectively use the insulin it produces leading to hyperglycemia [1].

In contrast to DM, tuberculosis (TB), is an infectious bacterial disease caused by *Mycobacterium tuberculosis*, which most commonly affects the lungs. People infected with TB bacteria have a 5–15% lifetime risk of falling ill with TB. However, persons with compromised immune systems, such as people living with HIV, malnutrition or diabetes, or people who use tobacco, have a much higher risk of falling ill [1].

WHO addresses the infectious disease like TB, and NCD like DM among the top 10 causes of death worldwide [1]. DM has been increasing in prevalence year after year, assumed to target mainly LMIC rather than high-income countries. In contrast to DM, TB occurs in every part of the world. However, the epidemic growth of DM especially occurs in developing countries, where TB is highly endemic, leading to increased mortality [2, 3].

Importantly, patients with TB who have DM also have worse TB treatment than those who do not have DM, which includes delayed conversion from positive to negative sputum cultures, higher risk of death during TB treatment and a higher risk of relapse after successfully completed treatment [2, 3].

Despite, TB in Nepal remains to be a major public health problem, minimal studies have been carried out regarding DM-TB comorbidity, which reveals the considerable prevalence of DM among TB patients (9.1%) [4]. Moreover, the SEAR experiences more unsettling problem of MDR-TB which worsens through comorbidities such as HIV and DM with TB, and jeopardizes the global objectives of ending TB by 2030 and eliminating TB by 2050 [2, 5].

The aim of the study is to determine the prevalence of diabetes and its associated risk factors among tuberculosis patients. The findings from the study will support the government for the collaborative framework of bidirectional screening of DM-TB comorbidity leading to early detection and treatment of both the disease simultaneously, decreasing the national prevalence of both DM and TB, and promoting the mental health and well- being of the people.

## Methods

A cross-sectional study of 6-month duration (1st September 2018 to 28th February 2019) was conducted at DOTS centers with laboratory facility of Morang, Eastern Nepal. Morang district consisted of 71 DOTS center including 20 with laboratory facility. Out of those 20, 8 DOTS center were randomly selected using lottery method.

### Sample size

All the TB patients aged 15 years and above who were registered under the National Tuberculosis Control Program and on treatment during the study period with completed intensive phase (2 months) treatment were included in the study. A sample size of 320 was calculated using a formula n = z^2^pq/e^2^ considering the prevalence from the study done in 2014 by Raghuraman et all in urban Puducherry and non-response rate of 10%.

### Data collection

A pretested semi-structured questionnaire based on WHO-STEPS instrument was used to collect the information related socio-demographic characteristics and behavioral risk factors [14]. Global Physical Activity Questionnaire (GPAQ) developed by WHO was used for assessing physical activity [14]. And, an excerpt from DASS-21 was used for the variable stress [15]. A face-to-face interview was taken by the researcher herself. Floor weighing scale for weight, and Linen tape for height, hip and waist circumference measurement were used for the anthropometric measurement. Fasting plasma glucose level and 2-h post-prandial glucose were estimated in the laboratory of the respective DOTS center by glucose- oxidase method, which used 2 ml of patient’s blood sample collected for each, by venipuncture. The blood sample was collected by the trained laboratory technician of the respective DOTS center.

On the first day, the purpose of the study was explained to the patient and participant information sheet written in Nepali (local language) was provided and informed written consent was obtained. The participants were asked to come the next day after overnight fasting for the test of fasting blood glucose (FBG) levels and were asked again to report after 2 h of having lunch for the 2-h post-prandial (PP) glucose test. The patients who had already been diagnosed for diabetes, details of the time of diagnosis and treatment taken was collected by asking medicine slip/ patient’s cards/ diagnosis slip to ensure the confirmation of the self-reported DM.

### Data analysis

Collected data were entered in Microsoft Excel 2016 and converted in Statistical Package for Social Science 11.5 version for statistical analysis. Descriptive analysis was done in the form of frequencies, percentages, mean, median and standard deviation. For inferential statistics, Chi-square test was applied to test the association between DM and others selected risk factors at 95% CI and level of significance as *P* = 0.05 for categorical data, while “students t-test” was used for continuous data. Variables with *P* value < 0.20 and with expected cell count not less than 5 were subjected to multivariate analysis (Binary Logistic Regression with category).

### Ethical consideration

Approval for this study was obtained following proposal review from the Institutional Review Committee (IRC) of BPKIHS (Code No: IRC/1265/018). Informed written consent was taken from respondents. Consent for publication has been taken from the author, co-authors and the respondents.

## Results

### Socio-demographic and socio-economic characteristics of the respondents

The study included 320 respondents, selected from 8 randomly chosen different DOTS centers of Morang, Eastern Nepal. The mean age of the respondents was 41.5 years with an abundance of male (70.0%). More respondent (70.9%) were Hindu by religion, and many (42.5%) were Janajati by their ethnicity. Likewise, many (62.2%) were currently married and had nuclear family (64.1%).

### Tuberculosis and diabetic status of the respondents

In this study, 38 (11.9%) respondents were found to have diabetes which includes 19 (50.0%) self-reported cases of diabetes. The remaining 19 (50.0%) were the new case diagnosed during the time of the study. Among those 19 self-reported diabetes cases, the study also reflected 9 (47.4%) to have a known history of diabetes prior to the tuberculosis treatment, with a median duration of five months. Despite, the low prevalence of diabetes (11.9%) in our study, there still exist a considerable portion (17.8%) of the respondents with impaired glucose and impaired fasting (17.8%). Table 1.

**Table 1:** Tuberculosis and Diabetic Status of the respondents

Characteristics	Frequency (*n* = 320)	Percent (%)
TB Type		
Sputum Positive	126	39.4%
Sputum Negative	142	44.4%
Extra Pulmonary	52	16.2%
TB Treatment Type		
New Case	285	89.1%
Relapse Case	35	10.9%
Diabetes-Tuberculosis Comorbidity		
TB Only	282	88.1%
TB-DM	38	11.9%
Impaired Glucose Tolerance (IGT)		
Yes	57	17.8%
No	263	82.2%
Impaired Fasting Glucose (IFG)		
Yes (Pre-Diabetic)	55	17.2%
No	265	82.8%
Diabetic Status		
Yes (Self-reported)	19	5.9%
No	47	14.7%
Don’t Know	254	79.4%
History of DM before TB Treatment (*n* = 19)		
Yes	9	47.4%
No	10	52.6%

### Behavioral characteristics

Among 320 respondents, 58 (18.1%) was a current user of tobacco products; 56 (96.6%) and 2 (3.4%) as daily and non-daily users of tobacco products respectively. This study finding also suggests that 179 (55.9%) and 83 (25.9%) were former user and never used tobacco products, respectively as categorized as non-user group in this study. Among those current users of tobacco products, many respondents 35 (60.3%) preferred smokeless tobacco. Nevertheless, further adjusting for different variables, the current user of tobacco products turns out to have a positive association with an odd of 2.247 times (OR = 2.247, 0.728–11.984).

However, the study findings reflect a small portion 14 (4.3%) of the respondents who currently drink the alcohol and 126 (39.4%) of each had drunk alcohol in 12 months and lifetime abstainer. Despite, a small portion (12.0%) of respondents constituted for who currently drinks alcohol (OR = 16.167, 4.262–61.322), after adjusting for other variables, they were found to have a very strong positive association for the development of diabetes with an odd of 12.307 times (OR = 12.307, 2.856–57.067).

The family history is the irreversible risk factor to diabetes is positively associated with diabetes, and the odds of getting diabetes is 2.463 times (OR = 2.463, 0.638–10.024).

Interestingly, in this study, neither the mean servings of fruits and vegetables nor the physical activity showed an independent association with diabetes. Tables 2 and 4.

**Table 2:** Behavioral Characteristics of the Respondents

Characteristics		TB only (*n* = 282)	TB-DM (*n* = 38)	Total (*n* = 320)	*P*-value
Use of Tobacco Product					
Current User (*n* = 58)	Daily	43 (97.7%)	13 (92.9%)	56 (96.6%)	0.001*^a^
	Non-Daily	1 (2.3%)	1 (7.1%)	2 (3.4%)	
	Total	44 (100.0%)	14 (100.0%)	58 (100.0%)	
Non-User (*n* = 262)	Former	168 (70.6%)	11 (45.8%)	179 (68.3%)	0.013*^a^
	Never	70 (29.4%)	13 (54.2%)	83 (31.7%)	
	Total	238 (100.0%)	24 (100.0%)	262 (100.0%)	
Alcohol Consumption					
Current Drinker (in 30 days)	Yes	7 (6.4%)	7 (53.8%)	14 (12.0%)	< 0.001*^a^
	No	97 (93.3%)	6 (46.2%)	103 (88.0%)	
Drank in past (12 months)	Yes	107 (63.3%)	13 (52.0%)	126 (61.9%)	0.277^a^
	No	62 (36.7%)	12 (48.0%)	74 (38.1%)	
Lifetime abstainer		113 (40.1%)	13 (34.2%)	126 (39.4%)	0.488^a^
Mean serving of fruits and vegetables per day		2.29 ± 0.63	2.18 ± 0.62	–	0.343^c^
Physical Activity					
Low Physical Activity		110 (39.0%)	13 (34.2%)	123 (38.4%)	0.178^a^
Moderate Physical Activity		148 (52.5%)	18 (47.4%)	166 (51.9%)	
High Physical Activity		24 (8.5%)	7 (18.4%)	31 (9.7%)	
Family History of DM					
Yes		71 (25.2%)	24 (63.2%)	95 (29.7%)	< 0.001^*b^
No		211 (74.8%)	14 (36.8%)	225 (70.3%)	

“*” Significant Association (P < 0.05)

*“a” Pearson Chi-Square*, *“b” Liner-by-Linear Association*, *“c” Independent Sample t-Test*

### Physical measures

The study reported abdominal obesity in more than half (55.2%) and one third (33.5%) of female and male, respectively and collectively, 40.0% of the total respondents in this study to have abdominal obesity. However, the bivariate analysis did not reflect an independent association of waist-to-hip (WHR) with diabetes.

With regard to the blood pressure of the respondents, the study reported a considerable portion (27.2%) of the respondents to be pre-hypertensive. However, the bivariate analysis did not reflect an independent association of blood pressure range with diabetes. Tables 3 and 4.

**Table 3:** Association of the Physical Measurements and diabetic status of the respondents

Characteristics		TB only (*n* = 282)	TB-DM (*n* = 38)	Total (*n* = 320)	*P*-value
BMI					
Underweight (< 18.5 kg/m^2^)		146 (51.8%)	22 (57.9%)	168 (52.5%)	0.738^a^
Ideal Weight (18.5–22.9 kg/m^2^)		105 (37.2%)	11 (28.9%%)	116 (36.2%)	
Overweight (≥23 kg/m^2^)		31 (11.0%)	5 (13.2%)	36 (11.3)	
Waist-to-Hip ratio					
Male	Obesity	65 (32.7%)	10 (40.0%)	75 (33.5%)	0.464^b^
	Normal	134 (67.3%)	15 (60.0%)	149 (66.5%)	
Female	Obesity	47 (56.6%)	6 (46.2%)	53 (55.2%)	0.480^b^
	Normal	36 (43.4%)	7 (53.8%)	43 (44.8%)	
Blood Pressure					
Normal		151 (53.5%)	23 (60.5%)	174 (54.4%)	0.879^a^
Pre-hypertension		81 (28.7%)	6 (15.8%)	87 (27.2%)	
Stage 1 Hypertension		47 (16.7%)	9 (23.7%)	56 (17.5%)	
Stage 2 Hypertension		3 (1.1%)	0	3 (0.9%)	
Mean Systolic Blood Pressure		117.23 ± 16.86	115.53 ± 18.41	–	0.562^c^
Mean Diastolic Blood Pressure		75.92 ± 10.30	74.21 ± 10.81	–	0.340^c^

*“a” Liner-by-Linear Association, “b” Pearson Chi-Square, “c” Independent Samples T Test*

**Table 4:** Predictors of the risk factors of Diabetes among the Tuberculosis Patients

Characteristics	Crude Odds Ratio (95% CI)	Adjusted Odds Ratio (95% CI) #
Family History		
Yes	5.095 (2.500–10.382)*	2.463 (0.638–10.024)
No	Reference	Reference
Current Use of Tobacco Products		
Yes	3.155 (1.515–6.570)*	2.247 (0.728–11.984)
No	Reference	Reference
Current Use of Alcohol (within 30 days)		
Yes	16.167 (4.262–61.322)*	12.307 (2.856–57.067)*
No	Reference	Reference
Stress Level		
Normal	Reference	Reference
Mild-Moderate	0.373 (0.139–0.999)*	1.930 (0.407–9.158)
Severe	0.823 (0.269–2.514)	3.816 (0.540–26.983)

“*” Significant Association (P < 0.05),

*“#” Adjusted for current tobacco user, current alcohol drinker, family history, and stress*

### Stress level

The present study finding reports two-third (63.1%) of the respondents having severe stress, and also reflects the positive association of diabetes with increasing level of stress. The odds for mild-moderate and severe level of stress of getting diabetes was 1.930 times (OR = 1.930, 0.407–9.158) and 3.816 times (OR = 3.816, 0.540–26.983), respectively compared to the normal stress level.

## Discussion

### Tuberculosis and diabetic status of the respondents

Out of 320 respondents selected from 8 randomly chosen different DOTS centers of Morang, Eastern Nepal, the prevalence of diabetes in the present study was found slightly higher (9.1%) than the study conducted in 2013 in Kathmandu [4] valley and China (6.3%) [6], however, lower enough than India [7, 8] and Pakistan [5] which reported the prevalence in the range of 20.0 to 30.0%. This was justified by the considerable portion of (42.5%) Janajati in the present study, as their culture and customs of drinking alcohol adjuvants the risk factor of diabetes among those people.

Despite, the low prevalence of diabetes (11.9%) in our study, there still exist a considerable portion (17.8%) of the respondents with impaired glucose and impaired fasting (17.8%). These findings are slightly lower than the study done in Tamil Nadu, India where diabetes and pre-diabetes were 25.3 and 24.5%, respectively [8]. However, the considerable portion of respondents with a family history of diabetes in both the studies; Tamil Nadu and our study justifies the pre-diabetic cases.

On the contrary to the study done in Kerala, India [9] which reported 55.6% of sputum positive cases, the present study reported only 39.4% of the sputum positive cases. This is somehow aligned by the fact that the present study included only the respondents who had completed the intensive phase (2 months) of the tuberculosis treatment regimen, unlike the study done in Kerala, India which had all the new and old cases under the tuberculosis treatment.

### Behavioral characteristics

Among the current user of tobacco products, many respondents 35 (60.3%) preferred smokeless tobacco which is comparatively higher (11.7%) than the study conducted in Tamil Nadu [8]. Tobacco product contains nicotine which has established evidence in insulin secretion and action, hence it justifies the association of the current user and non-user of tobacco products with diabetes-tuberculosis comorbidity [10].

Likewise, harmful use of alcohol is considered as the potential risk for onset of diabetes along with other chronic diseases by causing insulin resistance and pancreatic B-cell dysfunction [11].

However, the study findings reflect a small portion of 14 (4.3%) of the respondents who currently drinks the alcohol correlates the findings from the study done in Nepal in 2013 [4].

One of the irreversible risk factors of diabetes is family history, and as the findings from the study done in Puducherry [7] suggests, the odds of acquiring diabetes is 4.096 times (OR = 4.096, 1.730–9.698). This finding is higher, yet remains coherent in our study as well where the family history is positively associated with diabetes, and the odds of getting diabetes is 2.463 times (OR = 2.463, 0.638–10.024).

### Physical measures

Unlike Body Mass Index (BMI), waist-to-hip ratio is considered as the better predictor of abdominal obesity which correlates with the metabolic syndrome and other diseases [12]. The study reported collectively, 40.0% of the total respondents to have abdominal obesity which is higher than the findings from other studies done in Nepal [4] and India [8] where the abdominal obesity range from 5.2 to 34.9%.

With regard to the blood pressure of the respondents, the high blood pressure and diabetes have substantial overlap and frequently occurs simultaneously. Hypertension and diabetes share a common pathway and risk factors like obesity, physical inactivity, and unhealthy lifestyle.

### Stress level

Stress is associated with the release of cortisol, responsible for “flight and fight” response leading to a hyperglycemic state of the body. However, prolonged stress is a potential contributor to diabetes [13].

## Conclusion

To conclude, for every known case of diabetes, there are as many cases with impaired glucose tolerance and pre-diabetic, hence it may be inferred that active tuberculosis may be an alerting sign to unmask those pre-diabetic case for early diagnosis and treatment; shifting the focus on the bidirectional screening of tuberculosis and diabetes.

### Recommendation

As the study findings suggest current alcohol consumer as a significant predictor of Diabetes among the Tuberculosis patient on treatment, there exist the need to prioritize these populations in the adoption of bidirectional screening program for TB and DM. Furthermore, a prospective study, with the addition of the comparator group as general population would have strengthened the DM-TB comorbidity. Additionally, analyzing the blood sample by same trained laboratory personal would eliminate the inter-observer bias.

## Data Availability

The datasets generated and/or analyzed during the current study are not publicly available due to privacy policy of the human subject involved in the study but can be made available from the corresponding author on reasonable request.

## Abbreviations

DM: Diabetes Mellitus
DOTS: Direct Observed Treatment Short Course
HIV: Human Immune Deficiency Virus
LMIC: Low and Middle Income Country
MDR-TB: Multidrug-Resistant Tuberculosis
NCD: Non-Communicable Disease
SEAR: South-East Asian Region
TB: Tuberculosis
TB-DM: Tuberculosis Diabetes comorbidity
WHO: World Health Organization
